# Immunomodulatory Effects of Probiotics: A Novel Preventive Approach for the Control of Bovine Mastitis

**DOI:** 10.3390/microorganisms10112255

**Published:** 2022-11-14

**Authors:** A. K. M. Humayun Kober, Sudeb Saha, Md. Aminul Islam, Muhammad Shahid Riaz Rajoka, Kohtaro Fukuyama, Hisashi Aso, Julio Villena, Haruki Kitazawa

**Affiliations:** 1 Laboratory of Animal Food Function, Graduate School of Agricultural Science, Tohoku University, Sendai 980-8572, Japan; 2 Livestock Immunology Unit, International Education and Research Centre for Food and Agricultural Immunology, Graduate School of Agricultural Science, Tohoku University, Sendai 980-8572, Japan; 3 Department of Dairy and Poultry Science, Chittagong Veterinary and Animal Sciences University, Khulshi, Chittagong 4225, Bangladesh; 4 Department of Dairy Science, Sylhet Agricultural University, Sylhet 3100, Bangladesh; 5 Department of Medicine, Faculty of Veterinary Science, Bangladesh Agricultural University, Mymensingh 2202, Bangladesh; 6 Laboratory of Animal Health Science, Graduate School of Agricultural Science, Tohoku University, Sendai 980-8572, Japan; 7 The Cattle Museum, Maesawa, Oshu 029-4205, Japan; 8 Laboratory of Immunobiotechnology, Reference Centre for Lactobacilli (CERELA-CONICET), San Miguel de Tucuman 4000, Argentina

**Keywords:** probiotics, bovine mastitis, immunomodulation, bovine mammary epithelial cells

## Abstract

Bovine mastitis (BM) is one of the most common diseases of dairy cattle, causing economic and welfare problems in dairy farming worldwide. Because of the predominant bacterial etiology, the treatment of BM is mostly based on antibiotics. However, the antimicrobial resistance (AMR), treatment effectiveness, and the cost of mastitis at farm level are linked to limitations in the antibiotic therapy. These scenarios have prompted the quest for new preventive options, probiotics being one interesting alternative. This review article sought to provide an overview of the recent advances in the use of probiotics for the prevention and treatment of BM. The cellular and molecular interactions of beneficial microbes with mammary gland (MG) cells and the impact of these interactions in the immune responses to infections are revised. While most research has demonstrated that some probiotics strains can suppress mammary pathogens by competitive exclusion or the production of antimicrobial compounds, recent evidence suggest that other probiotic strains have a remarkable ability to modulate the response of MG to Toll-like receptor (TLR)-mediated inflammation. Immunomodulatory probiotics or immunobiotics can modulate the expression of negative regulators of TLR signaling in the MG epithelium, regulating the expression of pro-inflammatory cytokines and chemokines induced upon pathogen challenge. The scientific evidence revised here indicates that immunobiotics can have a beneficial role in MG immunobiology and therefore they can be used as a preventive strategy for the management of BM and AMR, the enhancement of animal and human health, and the improvement of dairy cow milk production.

## 1. Introduction

Bovine mastitis (BM) is one of the most prevalent and costly diseases of dairy cattle worldwide. BM refers to inflammation of the mammary gland (MG), which greatly affects milk production and impacts product quality, animal health and welfare, the economic profit of the dairy industry, and public health [1,2,3]. Numerous pathogens, including bacteria, viruses, and fungi can induce a variety of clinical symptoms, from asymptomatic sub-clinical infection to severe acute MG inflammation [4,5]. Acute inflammation brought on by the intense activation of immune cells by cytokines and chemokines production is one of the major clinical symptoms of MG infection [6,7]. The cure rate of mastitis depends on the species of mastitis-causing pathogens and the efficacy of antibiotics used, as well as the host immune status [8,9,10]. However, the irrational use of antibiotic medications frequently results in the emergence of antimicrobial resistance (AMR), which poses a serious threat to the wellbeing of dairy cattle. Additionally, AMR due to BM might pose serious risks to public health through the spread of AMR bacterial infections and drug residues through the consumption of raw milk from cows that have received antibiotic treatment [11]. Another major issue for the dairy sector is that the presence of antibiotic residues in milk can interfere with the fermentation process, particularly in cheese and yogurt production [1]. Due to both the low frequency of mastitis during lactation being cured and the high probability of AMR genes spread to pathogens by antibiotic therapy [12,13], it is necessary to review the traditional antibiotic-based treatment strategy and look for innovative and sustainable therapeutic alternatives. Herbal remedies, homeopathy, probiotics, genetic selection, and vaccination have been investigated as potential alternatives to antibiotics for the treatment or prevention of mastitis [14,15,16,17]. Compared to herbal therapy, homeopathy, and vaccination, it was found that probiotics may have a superior efficacy in terms of cost and effectiveness. In addition, the use of probiotics may reduce AMR, and it is environment friendly [18]. Thus, probiotics, particularly those with immunomodulatory capacities or immunobiotics, could be an interesting alternative to control or treat BM [3,19,20,21,22,23,24,25]. Immunobiotics have beneficial effects on human and animal health by modulating the host immune responses and are considered safe for human and healthy livestock production [19]. While most research has demonstrated that some probiotic strains can suppress mammary pathogens by competitive exclusion or the production of antimicrobial compounds, recent evidence suggest that other probiotic strains have a remarkable ability to modulate the response of MG to Toll-like receptor (TLR)-mediated inflammation. In this regard, using an originally established bovine mammary epithelial (BME) cell line, our group demonstrated that immunobiotic lactic acid bacteria (LAB) strains could be effectively used in the prevention of BM through the modulation of mammary epithelium immune responses [20]. The application of immunobiotics in the field of MG immunobiology is a novel concept. According to several research works, probiotics in dairy cows or breast-feeding women have reportedly inspired claims of usefulness for preventing or treating mastitis [26,27,28,29,30]. However, despite the amount of research being carried out on this topic, research is still needed to conclusively demonstrate the efficacy of immunobiotics and to clarify in detail their mechanisms of action. Considering the current understanding of host-bacterial interaction in MG mucosa, this review sought to explore whether the probiotic concept fits the MG biology, and if mastitis prevention or treatment is amenable to probiotic/immunobiotic intervention. A summary of scientific literature on the use of putative probiotics in mastitis trials is only taken into consideration here because the small number of studies did not allow meta-analysis. The review also explores the cellular and molecular interactions of immunobiotic LAB with bovine mammary epithelial cells and the transcriptional markers of mastitis, providing a novel preventive approach for the effective control of mastitis in dairy cows.

## 2. Global Economic Impact of Bovine Mastitis

BM causes enormous economic losses to the dairy industry. These economic losses are due to the treatment, production loss, changes in product quality, discarded milk, extra labor, culling, poor animal welfare, and the risk of other diseases [4]. Mastitis causes one of the biggest expenses for the dairy industry worldwide, including India [31], Bangladesh [32], Japan [33,34], Canada [35], the USA [36], Colombia [37], Netherlands [38], the United Kingdom [5], and Australia [39,40] for treatment and control. The cost or impact of mastitis on the economy varies by country, and even by region within a country, depending on market condition and milk quality regulations. Ultimately, BM contributes to potential financial losses to the farmer and the dairy industry. The anticipated losses to BM are related to 31% in milk production, 24% treatment costs, 18% wasted milk, 4% excess labor demand, and 23% premature culling [41,42]. A total annual cost due to BM has been estimated as USD 19.7 to USD 32 billion in the global dairy industries [36]. Annual economic losses due to BM are projected to be USD 2 billion in the United States [36], USD 0.31 billion in Canada [35], USD 0.8 billion in Colombia [37], USD 1.3 billion in Australia [39,40], USD ~1.55 billion in the EU [39], USD ~0.77 billion in Japan [33,34], USD ~0.002 billion in Bangladesh [32], and USD ~0.8 billion in India [31]. A single case of clinical mastitis may lead to an economic loss that ranges between USD 128 and USD 444 [41,42]. According to these data, mastitis has a significant economic impact on dairy cattle production.

## 3. Prevention and Control Strategies of Bovine Mastitis

Currently, most BM management strategies focus on prevention during milking by following hygienic milking practices and a good management system. Other alternative approaches for the control of mastitis include vaccination, antibiotics, immunotherapy, bacteriocins, bacteriophages, antimicrobial peptides, stem cell therapy, native secretory factors, diet, dry cow and lactation therapy, genetic selection, botanicals, nanoparticle technology-based therapy, and probiotics [18]. The most widely utilized techniques to treat mastitis are antibiotic therapy and vaccination, which are not always effective in the control of BM. Extensive and uncontrolled use of antibiotics for the treatment along with the induction and spread of AMR in mastitis has led to reduced responsiveness to antibiotic therapy [43,44]. On the other hand, vaccination has limited effectiveness against BM because of the variety of microorganisms involved in its development [24,45,46]. Even though there are several commercial vaccines available, the majority do not provide complete protection, and are also expensive, limiting their extensive application [18,47]. Thus, alternative tactics are required to efficiently manage mastitis. 

**Figure 1:**
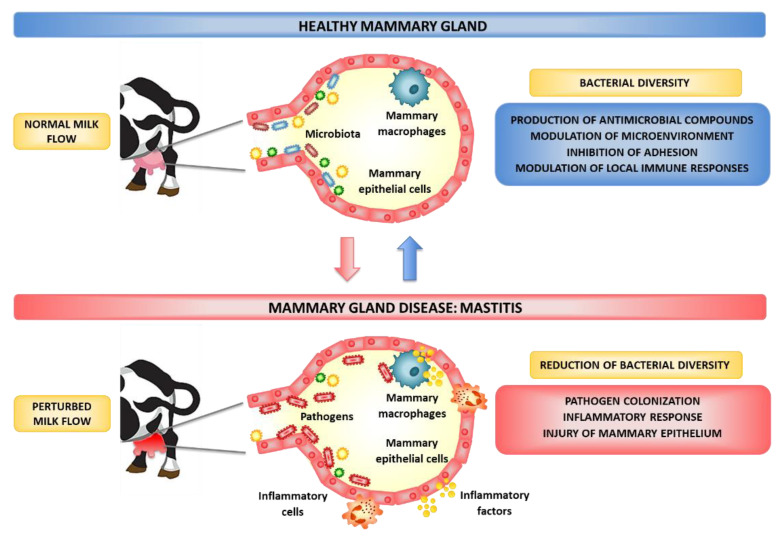
Effect of bovine mammary gland microbiota on the susceptibility to infections.

The high-throughput sequencing of bacterial 16S rRNA genes performed in the past few years has demonstrated the existence of bovine milk and MG microbiota [48]. In fact, a diverse range of opportunistic and commensal bacteria can inhabit the intramammary ecosystem [49,50]. Metagenomic investigations have demonstrated diverse bacterial groups in MG and milk samples obtained from non-mastitis quarters of dairy cows. Furthermore, there is growing evidence that the reduction of the diversity or the alteration of MG significantly augment the susceptibility to BM [49,50]. It is postulated that MG commensal bacteria help the bovine host to resist the pathogens’ colonization by several mechanisms, including competition for nutrients, conditioning the microecosystem to disfavor pathogen growth through organic acid production, the synthesis of antimicrobial compounds, the inhibition of adhesion to epithelial surfaces, and the regulation of MG epithelium immunity [49,50] (Figure 1). Recent studies revealed that probiotics may be used as a novel tool to treat or prevent BM while also addressing the issue of emerging AMR [3,20,27]. One innovative preventive strategy for the management of BM is the use of probiotics with immunomodulatory capacities or immunobiotics.

## 4. In Vivo Studies of Probiotics for the Prevention or Treatment of Bovine Mastitis

The microorganisms that have been used as probiotics for the prevention or treatment of BM are presented in Table 1. Some studies have evaluated the ability of probiotics to protect against BM using mice models. *L. lactis* LMG 7930 was shown to possess antagonistic effects against ruminant mastitis-causing pathogens [51]. Campeiro et al., 2017 [51] conducted an in-depth investigation of the mammary gland immune response induced by intramammary inoculum of a live culture of the LMG 7930 strain using the mouse mastitis model. The work reported that *L. lactis* LMG 7930 injected into the mouse inguinal glands before the injection of *Staphylococcus chromogenes,* decreased pathogen loads by improving the innate immune response. More recently, it was shown that *Enterococcus mundtii* H81, originally isolated from the milk of healthy dairy cows, injected to the nipple duct before *S. aureus* infection reduced neutrophil infiltration and *TNF*-α and *IL-1β* production, as well as the phosphorylation of p65 nuclear transcription factor kappa B (NF-κB) and IκB [52].

**Figure 2:**
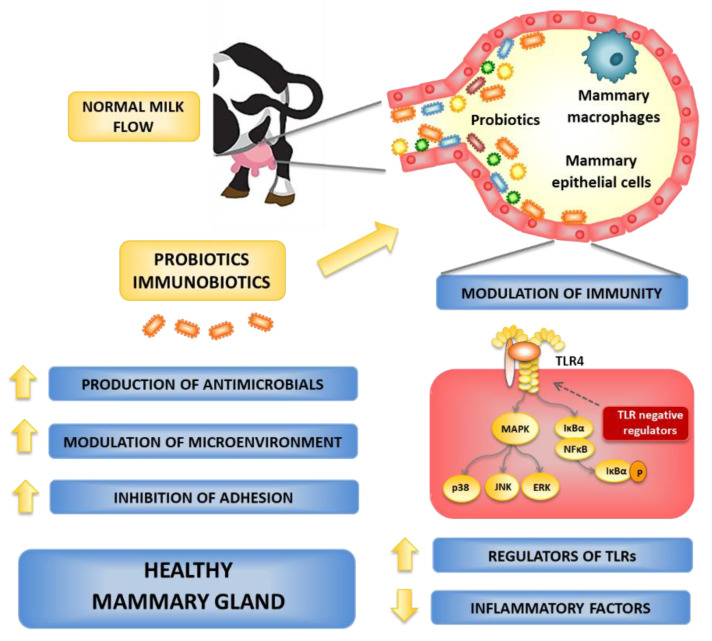
Effect of probiotic and immunobiotic microorganisms on the susceptibility to bovine mammary gland infections.

To combat mastitis, probiotics have also been administered to cows by the oral route or through an intramammary infusion. When LAB such as *L. lactis* are infused into the MG quarters, they induce significant increases in the recruitment of neutrophils and lymphocytes to the udder [17]. The administration of live culture of *L. lactis* DPC3147 into the MG enhance the innate immune response [17]. The expression of *IL*-*1β* and *IL-8* genes were markedly increased in DPC3147-treated animals, with the highest expression corresponding to high somatic cell count in bovine mammary secretion [17,53]. Moreover, live cultures of *L. lactis* DPC3147 showed the same potential as antibiotic therapy for the eradication of persistent subclinical mastitis [17]. In addition, different strains of *Lactobacillus*, *Bacillus*, *Bifidobacteria*, and *Enterococcus* could reduce inflammation of the MG and control the milk microorganisms in dairy cows with mastitis [48]. Peng et al. (2013) [7] found that ultrasonicated *Enterococcus faecium* SF68 is compatible with cow MG and is an enhancer of innate immunity by accelerating neutrophil chemotaxis immediately following milking and lowering the somatic cell count (SCC) in milk. The treatment with *Bifidobacterium breve* and *L. lactis* subsp. *lactis* LMG 7930 reduces the infection rate of subclinical mastitis and SCC in milk [54,55]. Furthermore, the *B. breve* and *L. lactis* DPC3147 used in intramammary infusion lessens chronic subclinical mastitis in cows [51,54]. Another study observed that the intramammary infusion of *L. lactis* subsp. *lactis* CRL1655 and *Lactobacillus perolens* CRL1724 can mitigate the mastitis and lower SCC in milk by inducing a rise in IgG levels in blood and milk [29]. Animals that had received *L. lactis* CRL1655 and *L. perolens* CRL1724 infusions did not show any clinical symptoms related to MG infection. Of note, the blood serum of lactobacilli-treated animals had markedly enhanced opsonophagocytic activity against *Staphylococcus* spp. and improved expression of the proinflammatory cytokines *IL-1β*, *IL-8*, and *TNF-α* [29]. Intramammary treatment with inactivated cultures of *Lacticaseibacillus rhamnosus* GG to quarters naturally affected by subclinical mastitis has a transient pro-inflammatory activity assessed by low SCC and is capable of modifying the microbiota of milk [55]. These findings revealed that different probiotic strains have the potential to prevent mastitis by antagonizing pathogens, relieving MG inflammation, and regulating milk microorganisms (Figure 2).

On the other hand, there are fewer studies that have evaluated the effect of orally administered probiotics for improving mastitis in animals. It was observed that the addition in the feed of the marine-derived *Bacillus amyloliquefaciens* GB-9 enhanced immunity and protected against mastitis in Saanen dairy goats [56]. It was also demonstrated that the feeding of Holstein cows with supplemental *Saccharomyces cerevisiae* and *L. lactis* reduced MG inflammation and the proportion of microorganisms with the ability to cause BM, including *Enterococcus* and *Streptococcus* [53]. Similarly, the addition to the diet of a symbiotic formulated with medicinal plants (*Taraxacum officinalis* and *Zingiber officinalis*, and *Lactobacillus* and *S. cerevisiae*) significantly reduced the prevalence of subclinical mastitis [57]. In addition, supplements of *S. cerevisiae* improved the calcium level in blood [58] and tended to reduce mastitis. We evaluated the capacity of *Bacillus subtilis* C-3102, which has been extensively used as a probiotic feed additive for livestock, to impact on dairy cows with a previous history of mastitis [59]. The prophylactic administration of the C-3102 strain significantly reduced the mean SCC in milk. When blood immune cells were analyzed, no differences were found in the levels of granulocytes, monocytes, and B cells between control and C-3102-treated animals. However, cows receiving *B. subtilis* C-3102 had an increased proportion of CD4^+^ T cells and lower levels of the inflammatory WC1^+^γδ^+^ T cells and CD8^+^γδ^+^ T cells. In addition, bovine peripheral blood dendritic cells were analyzed, and it was found that the C-3102 treatment increased CD11c^+^CD172a^high^ populations, while it did not affect the levels of CD11c^+^CD172a^dim^ dendritic cells [59]. Therefore, the oral ingestion of probiotics has the potential to offer beneficial effects beyond intestinal health, but the mechanism underlying these effects in distal mucosal sites such as the MG in the bovine host remains largely unknown.

**Table 1:** Summary of in vivo studies of probiotics against bovine mastitis.

Species	Administration Route	In-Vivo Model	Mechanism of Probiotics against Mastitis	References
*L. lactis* LMG 7930	Injection through inguinal glands	CD-1 mice	*Staphylococcus chromogenes* ↓ *IL-1β* ↑ *TNF*-α ↑	[51]
*E. mundtii* H81	Intramammary infusion	BALB/c mice	Pathogens ↓ *NF-κB* ↓ Inflammatory response ↓	[52]
*L. lactis* DPC3147	Infusion via streak canal	Holstein–Friesian cows	Lymphocytes ↑ Neutrophils ↑ Acute phase protein haptaglobin ↑ Milk amyloid A ↑	[17]
*L. lactis* DPC3147	Intramammary infusion	Holstein–Friesian, New Zealand Friesian, Norwegian Red, Normande and Montebelliards cows	Pathogens ↓ Intramammary response ↑	[29]
*L. lactis* DPC 3147	Intramammary infusion	Holstein Friesian cows	*IL-8* ↑ Somatic cell count ↓	[60]
*L. rhamnosus* GG	Intramammary inoculation	Multiparous water buffaloes (*Bubalus bubalis*)	Leukocytes ↑ *Pseudomonas* ↓ Somatic cell count ↓	[55]
*E. faecium* SF68	Intramammary infusion	Holstein cows	Innate immunity ↑ Metalloproteinase 9 ↑ Neutrophil infiltration ↓	[7]
*B. breve*	Intramammary infusion	Holstein cows	Pathogens ↓ Innate immune response ↑ Somatic cell counts ↓	[61]
*B. breve*	Intramammary Infusion	Holstein cows	Pathogens ↓ Innate immune response ↑	[54]
*L. lactis* subsp. *lactis* CRL1655; *L. perolens* CRL1724	Intramammary infusion	Holstein cows	Pathogens ↓ Innate immune response ↑ Somatic cell counts ↓	[30]
*Saccharomyces cerevisiae* and *Lactobacillus*	Oral feed	Fleckvieh cows	Prevalence of subclinical mastitis ↓	[58]
*Saccharomyces cerevisiae* and *L. lactis*	Oral feed	Holstein cows	Mammary gland inflammation ↓ *Enterococcus* ↓ *Streptococcus* ↓ *Lactococcus* ↑	[48]
*Bacillus subtilis* C-3102	Oral feed	Holstein cows	Inflammation ↓ Blood CD4^+^ T cells ↑ Blood CD11c^+^CD172a^high^ dendritic cells ↑ Blood WC1^+^γδ^+^ T ↓ Blood CD8^+^γδ^+^ T cells ↓	[59]

Abbreviations: ↑, increased; ↓, decreased.

## 5. In Vitro Studies of Probiotics for the Prevention or Treatment of Bovine Mastitis

The use of probiotics in animal trials has been shown to improve udder health, reduce udder inflammation by lowering the pathogen load, and lower the SCC in milk, according to the in vivo research described above (Table 1). However, there is still no clarity on the precise underlying mechanisms of the function of probiotics in the prevention of BM in cattle due to the limitation of adequate mammary experimental systems related to the bovine host. Several research groups have performed in vitro studies to evaluate the effect of probiotics against predominant mastitis-causing pathogens [62,63,64,65,66,67,68,69]. It was shown that several microorganisms can beneficially modulate parameters related to the control or prevention of BM (Table 2). Studies have reported the ability of potential probiotic strains to induce inhibition and antagonistic activity against relevant BM pathogens, including *S. aureus*, *S. agalactiae*, *S. haemolyticus*, *S. simulans*, *S. vitulinus*, *Listeria innocua*, and *L. monocytogenes.*

Considering that the mammary epithelium acts as a first line of defense against pathogens causing BM, several studies focused on the in vitro evaluation of potential probiotic strains using bovine mammary epithelial cells (Table 2). The use of primary cells for in vitro investigation has the benefits of reflecting appropriate mitogenic responses as well as preserved physiological functions, including those related to the generation of immune responses [70]. However, the isolation of epithelial cells from MG tissue is costly, difficult, and allows only single and short-term experiments. For this reason, the use of cell lines has advantages over primary cultures because of their replicate capability by numerous passages and the retention of reasonably constant cellular characteristics [3]. Different types of cell lines, including bovine mammary epithelial cells and bovine teat canal epithelial cells, have been used to evaluate the efficacy of potential probiotic strains against mastitis pathogens (Table 2). The teat canal of a cow is a longitudinally folded cylinder-shaped body covered with an epithelial cell layer, which has contact with the teat lumen and the environment [12,30]. Thus, some studies concentrated in BTCEC cells as in vitro models. It was shown that LAB strains including *L. lactis* subsp. *lactis* CRL 1655 can adhere to teat canal epithelial cells and inhibit the growth of *S. aureus*, *S. epidermidis*, *S. xylosus*, *S. uberis*, *S. agalactiae*, and *E. coli* [30,71]. Similarly, it was demonstrated that LAB strains may interfere with the adhesion of *S. aureus* to the MDA-MB-231 [59] and bMEC [72] cell lines as well as decrease the internalization of *S*. *agalactiae*, *S. intermedius*, and *S. aureus* in BME-UV1 cells [56].

**Table 2:** Summary of in vitro studies of probiotics against bovine mastitis.

Species/Strains	Experimental Mode	Probiotics Effects	References
*L. bulgaricus*	In vitro (SDS-PAGE)	Inhibition *S. aureus* and *S. agalactiae* by bacteriocin production	[62]
*L. acidophilus* DSM 20079, *L. plantarum* ATCC 8014, *L. casei* ATCC 39392, *L. reuteri* ATCC 23272	In vitro (Well diffusion and Co-culture)	Inhibition and antagonistic activity against *S. aureus*	[63]
*L. helveticus*	In vitro (Well diffusion and Co-culture)	Antimicrobial activity against *S. aureus*, *S. haemolyticus*, *S. simulans*, *S. vitulinus*	[64]
*B. amyloliquefaciens*, *B. cereus, B. licheniformis*, *B. subtilis* ATCC 21332	In vitro (Agar diffusion)	Inhibition of Gram-positive bacteria	[65]
*L. sakei* EIR/BG-1	In vitro (Well diffusion)	Antimicrobial and antibiofilm activity against *S. agalactiae*, *S. dysgalactiae subsp. dysgalactiae*, and methicillin resistant *S. aureus*	[66]
*L. rhamnosus* ATCC 7469, *L. plantarum* 2/37	In vitro (Culture based method)	Antimicrobial activity against *S. aureus*, *S. xylosus*, and *S. epidermidis* by biofilm production	[67]
*L. plantarum*	In vitro (Phage mixture)	Antimicrobial activity against *S. aureus*	[68]
*Enterococcus hirae* CRL 1842, *E. hirae* B6.1B, *Enterococcus hirae* CRL 1846, *Enterococcus hirae* CRL 1847, *Enterococcus hirae* CRL 1848, *Enterococcus hirae* CRL 1837, *Enterococcus hirae* CRL 1834, *Enterococcus hirae* CRL 1835	In vitro (Diffusion plate technique)	Inhibition of *L. innocua*, *L. monocytogenes*, and *S. dysgalactiae* by bacteriocin production	[69]
*L. paracasei*, *L. plantarum*, *L. lactis*, *L. rhamnosus*	BTCEC	Adhesion to teat canal epithelial cells and inhibition of the growth of *S. aureus*, *S. epidermidis*, *S. xylosus*, *S. uberis*, *S. agalactiae*, and *E. coli*	[30]
*L. perolens*, *L. lactis* subsp. *Lactis* CRL 1655	BTCEC	Adhesion to teat canal epithelial cells and inhibition of the growth of *S. dysgalactiae* and *S. aureus*	[71]
*L. casei* BL23	bMECs	Anti-inflammatory properties and inhibition of the internalization of *S. aureus*	[73]
*L. lactis* V7	bMECs	Modulation of CXCL8 production and inhibition of cell invasion by *S. aureus* and *E. coli*	[74]
*L. casei* CIRM-BIA 667	bMECs	Adhesion to teat canal epithelial cells and inhibition of the growth of *S. aureus*	[72]
*L. brevis* 1595, *L. brevis* 1597, *L. plantarum* 1610, *L. casei* 1542, *L. lactis* 1596, *L. garvieae* 1605	bMECs	Anti-inflammatory properties (reduction of *IL-8*) against *E. coli*	[9]
*L. gasseri* LA806	bMECs	Reduction of proinflammatory cytokines (*IL-8*, *IL-6*, *IL-1α*, *TNF-α*) and prevention of *S. aureus* colonization	[13]
*P. stilesii* HOL36L1, *L. lactis* GIRO4S8, *W. paramesenteroides* GIR46L4, *W. confusa* GIR48L1, *W. cibaria* GIRO27L2, *L. plantarum* GUZ3L2, *L. paracasei* GIR53L1, *S. lutetiensis* HOL36L2	MDA-MB-231	Interference with adhesion and inhibition of *S. aureus*	[75]
*L. lactis* LMG 7930	BME-UV1	Reduction of the internalization of *S. aureus*, *S*. *agalactiae*, *S. aureus*, S. *chromogenes*, *S. epidermidis*, *S. intermedius*, and *L. cremoris*	[51]
*L. lactis* LL11	BME-UV1	Reduction of *TNF*-α against mastitis pathogen	[76]
*L. rhamnosus* GR-1	bMECs	Ameliorates *E. coli* induced Inflammation	[77]
*L. acidophilus* CRL2074	BME	Reduction of proinflammatory cytokines and chemokines (*IL-1α*, *IL-1β*, *MCP-1*, *IL-8*, *CXCL3*) induced by *E. coli* derived LPS	[3]

Abbreviations: bovine mammary epithelial cells (BME, BME-UV1, bMEC, bMECs); bovine teat canal epithelial cells (BTCEC).

When pathogenic bacteria enter the udder lumen via the teat canal, they interact with bovine mammary epithelial cells to establish colonization. This microbe invasion is sensed by the host through pattern recognition receptors (PRRs), such as the Toll-like receptors (TLRs) expressed in bovine mammary epithelial cells that detect microbial associated molecular patterns (MAMPs). This pathogen–epithelial cell crosstalk induces the generation of a local inflammatory response [78]. Some studies have also focused on the ability of potential probiotic strains to modulate the immune responses of bovine mammary epithelial cells, particularly in their capacity to beneficially regulate the production of inflammatory cytokines and chemokines (Table 2). It was observed that the treatment of bMEC cells with *L. lactis* V7 [57], *L. gasseri* LA806 [13], and other LAB strains [9] differentially modulated the production of inflammatory factors in response to *S. aureus* or *E. coli* challenges. Similarly, *L. lactis* LL11 was shown to reduce *TNF-α* production in BME-UV1 cells after challenge with BM pathogens [32].

Considering that *E. coli* infection in the bovine mammary gland often results in clinical mastitis, which is characterized by acute symptoms of inflammation [79], and that Gram-negative bacteria induce inflammatory responses through lipopolysaccharide (LPS) [80], some studies focused in the TLR4 signaling pathway in the MG epithelium. It was reported that the challenge of primary bovine mammary epithelial cells with *E. coli* induce a remarkable inflammatory response characterized by the increased expression of *TLR4*, *NOD1*, *NOD2*, pyrin domain-containing protein 3 (*NLRP3*), and the serine protease caspase 1 [77]. These changes were associated with the up regulation of *TNF-α*, *IL-1β*, *IL-6*, *IL-8*, and *IL-18.* Of note, the treatment of MG cells with *L. rhamnosus* GR-1 significantly reduced the expression of all inflammatory genes. Furthermore, the authors reported an improved expression of *IL-10* in bMECs treated with the GR-1 strain. Our group developed a bovine mammary epithelial cell line (BME cells) for the evaluation of probiotics/immunobiotics in terms of their capacity to modulate pro- and anti-inflammatory responses against pathogen invasion (2002). We demonstrated that TLR4 is strongly expressed in BME cells and that LPS stimulation induces a remarkable transcriptomic response characterized by the up regulation of inflammatory genes [27]. In our experience, TLR4 activation in BME cells resulted in the expression of *IL-1α*, *IL-1β*, *MCP-1*, *CXCL2*, *CXCL3*, *CXCL5*, *CXCL8*, *CXCL9*, and *C6*, inflammatory genes that were not significantly modified after the activation of the TLR2 signaling pathway [27]. Our results showed that the in vitro BME cell system could be of value for the evaluation of immunomodulatory treatments aimed at beneficially modulating inflammation in the bovine mammary gland. Also, the findings of Rose et al. (2017) [81] corroborate to our study. In this regard, using several LAB strains isolated from the feedlot cattle environment [82], we studied the capacity of potential immunobiotic strains to modulate TLR4-mediated inflammation in BME cells. We found a strain dependent capacity of LAB to modulate the innate immune response in bovine mammary epithelial cells when BME cells were treated with lactobacilli before challenge with LPS. *L. acidophilus* CRL2074 decreased the expression of *IL-1α*, *IL-1β*, *MCP-1*, *IL-8*, and *CXCL3*, while *L. rhamnosus* CRL2084 diminished the expression of *IL-1β*, *MCP-1*, and *IL-8* in BME cells challenged with *E. coli* derived LPS [3]. Of note, our study was the first in demonstrating that immunomodulatory lactobacilli may exert their beneficial effects on LPS-induced inflammation by differentially modulating the expression of negative regulators of the TLR4-signaling pathway in bovine mammary epithelial cells. In fact, *L. acidophilus* CRL2074 was able to upregulate *A20*, *SIGIRR*, and *Tollip* expression, whereas *L. rhamnosus* CRL2084 only upregulated the expression of *Tollip*, which was consistent with its lower capacity to modulate inflammatory genes in BME cells [3]. The information obtained in our in vitro studies allows us to propose a potential mechanism of action to explain the immunomodulatory effect of LAB strains such as *L. acidophilus* CRL2074 (Figure 2). Immunobiotic lactobacilli would interact with BME cells and be recognized by immune receptors such as TLR2, inducing the up regulation of negative regulators of TLR-signaling. The subsequent challenge of BME cells with LPS or Gram-negative pathogens such as *E. coli* would occur in the presence of the transcriptomic changes already induced by lactobacilli, which would differentially regulate TLR4-MyD88-NF-kB signaling cascade, reducing the secretion of inflammatory cytokines and chemokines. Further studies are necessary to find the precise bacterial molecules and immune receptors involved in the induction of TLR negative regulators in the MG epithelium by immunobiotic bacteria, which would also explain the differences between the strains.

## 6. Possible Routes for Probiotics to Control the Bovine Mastitis

In practical terms, probiotics can be applied to bovine hosts through different routes, including intramammary, topical, or orally.

### 6.1. Local Administration of Probiotics

The intramammary administration of probiotics would have the benefit to act at different levels, as follows: (a) induce the colonization of probiotic strains that would locally produce substances with the capacity to inhibit the growth of pathogens as well as interfere with their adhesion, and/or (b) modulate the local mucosal immune response, impacting both innate and adaptive immunity (Figure 2). Innate immune mechanisms predominate in the early stage of infection in the MG, and they are mediated not only by epithelial cells but also by local immune cells such as macrophages. These phagocytes are in the alveoli and can rapidly recognize and phagocytize bacteria that enter the MG cistern. In response, activated macrophages release pro-inflammatory cytokines and chemokines that induce the recruitment and activation of immune cells, including neutrophils and natural killer cells (NK) that help to control the infection [3,20,27]. In vitro models based on macrophages from bovine MG are not available and therefore it is not possible to assess the impact of intramammary administered probiotics on the ability of these immune cells to respond to pathogens. The development of in vitro models based on macrophages from the bovine mammary mucosa would be of great value, not only to predict the impact of probiotics on the local innate immune responses but also on adaptive immunity, considering that the activated macrophages can modulate the responses mediated by B and T cells through their membrane molecules and the production of biological mediators.

Another interesting line of research that has been raised in recent years is the topical administration of probiotics and the use of bacterial colonizers at the teat apex of *MG.* As mentioned earlier, the port of entry for most MG pathogens is the teat canal. The function of the teat sphincter muscle is to keep the orifice closed and thereby isolate the interior of the MG. This means that any damage to this structure is related to an increase in the incidence of mastitis. The teat canal is lined with keratin, which provides an additional physical barrier, preventing bacterial migration towards the MG cistern [83,84]. Esterified and non-esterified fatty acids (myristic, palmitoleic, and linoleic) function as bacteriostatics and are associated with keratin. Moreover, certain cationic proteins associated with keratin can bind to pathogens, increasing their susceptibility to osmolarity changes [83,85].

Of note, the teat skin, particularly the teat apex proximal to the entrance of the teat canal, and the teat canal itself are colonized by microbiota. Recent research has begun to determine the composition of this microbial population and evaluate its impact on the resistance to infections. It was shown that one member of the microbiota is *Corynebacterium bovis*. This species of bacterium was considered a minor mastitis pathogen with limited clinical significance [86], while more recent studies have suggested that it is part of the udder core microbiota, with a potential protective role against dysbiosis [87]. *C. bovis* has become of interest because quarters harboring this bacterium are less likely to become infected with other more severe pathogens. Compared to uninfected mammary quarters, those that had shed *C. bovis* were more resistant to an experimental intramammary challenge with *S. aureus*. However, they were more sensitive to a challenge with *S. agalactiae* [88]. It was also shown that bacteriocins produced by certain *Corynebacterium* species colonizing the teat canals may inhibit the growth of major mastitis pathogens [89]. Thus, the colonization of cattle teats with the members of teat skin microbiota might be a potential tool for the prevention of bovine mastitis. A similar approach is being explored with members of the respiratory commensal microbiota, including strains of the species *Corynebacterium pseudodiphtheriticum* [90,91] and *Dolosigranulum pigrum* [92], which are used to effectively improve resistance against the bacterial and viral pathogens of the respiratory tract. Further studies are needed to step forward in the positioning of certain strains of the local microbiota as next-generation probiotics for MG.

### 6.2. Oral Administration of Probiotics

As mentioned before, the oral ingestion of probiotics has the potential to offer beneficial effects beyond intestinal health, positively impacting bovine MG. Few studies have evaluated the effect of orally administered probiotics on the BM (Table 1). In addition to those studies, it was shown that the oral administration of *L. casei* Zhang and *L. plantarum* P-8 significantly reduced SCC and augmented the production of milk as well as the concentrations of milk IgG, lactoferrin, and lysozyme [93]. Although pathogens associated with mastitis or parameters that evaluate the local inflammatory response were not studied in this work, the results gave indirect evidence of the beneficial effect of orally administered probiotics on MG. The mechanisms involved in the beneficial effects induced by orally administered probiotics/immunobiotics in the bovine host remains largely unknown. Some studies carried out in humans and mice could give clues to these mechanisms. Two hypotheses, not mutually exclusive, have been proposed to explain the effect in MG induced by orally administered probiotics: (a) entero-mammary bacterial migration, and (b) the modulation of the common mucosal immune system.

The concept of entero-mammary bacterial translocation involves the transport of live bacteria from the gut to the MG [94], and studies have suggested that antigen presenting cells (dendritic cells and macrophages) play an important role in this process [95]. It was demonstrated that the intestinal microbiota obtained from cows with mastitis transferred to Germ-free mice via the fecal microbiome transplantation method induced the development of mastitis in recipient mice [96]. Microbiota from cows with mastitis enhanced the expression of several inflammatory markers in murine mammary glands, including *NF-κB*, *MAPK-ERK*, *MAPK-p38*, *MAPK-JNK*, and *STAT3*, and the production of *IL-6* and *TNF-α*. Of note, this effect was not observed when the intestinal microbiota were obtained from healthy cows. Interestingly, when *L. casei* Zhang was used as a supplement in the intestinal microbiota from cows with mastitis, the induction of mastitis in mice and the inflammatory response were significantly reduced [96]. Similarly, other recent works demonstrated the capacity of the fecal transfer of the intestinal microbiota from cows with mastitis to induce MG disease in Germ-free mice [97]. The studies suggest that intestinal microorganisms, including orally administered probiotics, could be transported to the MG. However, whether or not the two ecological niches are inter-related to undergo constant microbial exchanges still need to experimentally proven.

On the other hand, it was demonstrated that intestinal microbiota and orally administered probiotics can regulate the so called “common mucosal immune system”, exerting beneficial effects not only in the intestinal mucosa, but also in distal mucosal sites [98]. Of note, most scientific works investigating the capacity of orally administered probiotics to modulate immune responses in distal mucosal sites have focused mainly on the respiratory tract [98,99]. Research work demonstrated that probiotics could induce the mobilization of B and T cells from the intestinal mucosa to the respiratory tract. These immune cells homed in the respiratory mucosa produce immune factors such as IgA and cytokines, including *IFN-γ* and *IL-10*, which regulate the local population of immune and non-immune cells, enhancing their ability to respond to pathogens and protecting against inflammatory damage [100]. It is speculated that probiotics could exert a similar effect on MG immunity. It was demonstrated that B cells from the maternal gut can migrate to the MG during late pregnancy and lactation [101]. The mobilized B cells produce IgA in the MG mucosa, helping to improve the protection not only locally but in addition to the nursing child. Pioneer studies by Ahlstedt et al. (1975) [102] suggested that the IgA-secreting B cells homed in the MG originated in the gastrointestinal tract. Furthermore, it was shown that orally administered non-pathogenic *E. coli* to pregnant women resulted in a significant enhancement of IgA-secreting B cells specific against *E. coli* antigens in the colostrum [103]. The treatment of rats with *L. fermentum* CECT5716 during pregnancy and lactation improved the milk composition and the levels of IgA [104]. Similarly, it was found that the oral administration of the probiotic strain *L. casei* CRL431 to mice increased the levels of IgA-producing B cells in MG, but it did not affect the numbers of CD4^+^ or CD8^+^ T cells [105]. It has also been postulated that the modulation of systemic immune responses by orally administered probiotics could impact MG immunity. The oral administration of the CRL431 strain to mice differentially modulate the production of inflammatory and regulatory cytokines, improving the immune response to breast cancer cells [106]. In line with these studies, we postulated that orally administered *B. subtilis* C-3102 to cows activates blood dendritic cells, which then migrate into the MG where they can help to regulate inflammation via the induction of Treg cells [59]. These studies indicate that the modulation of the common mucosal immune system by probiotics has the potential to beneficially modulate immunity in MG, although further research should explore the effects and mechanisms in details in the bovine host.

The few studies conducted so far with orally administered probiotics postulate two mechanisms to explain the beneficial effects as mentioned above. Both the stimulation of the common mucosal immune system and the transport of bacteria from the intestine to the MG involve an interaction of microorganisms with cells of the immune system, giving the latter a relevant role in the effect of probiotics. Further cellular and molecular studies focused on the interaction between probiotics and bovine intestinal immune cells, and the impact of this interaction on the immunobiology of the MG, are of fundamental importance, not only to elucidate the mechanisms of action but also to improve the selection and implementation of probiotic feeds intended to increase resistance to BM.

## 7. Conclusions

In the present review, we provided a general overview of the research works demonstrating the capacity of probiotics to improve the resistance to mastitis in the bovine host. Probiotic microorganisms may influence susceptibility to MG infection through different mechanisms, including microbe–microbe interactions and modulation of immune responses. We highlight the potential of immunobiotic strains with the capacity to modulate the immune responses in bovine mammary epithelial cells as a safe and effective alternative to combat BM. Research papers provide strong evidence for the benefits of local application (intramammary or topical) of probiotics, while effects mediated by orally administered beneficial microorganisms need further investigation. Immunobiotic LAB can be used as an efficient immunobiotechnological tool to help in controlling AMR, which ultimately will lead to the development of the drug-independent healthy production of dairy cows.

## Data Availability

Not applicable.
